# Generative artificial intelligence in lung cancer care: current applications, challenges, and future directions

**DOI:** 10.3389/fonc.2026.1915669

**Published:** 2026-09-11

**Authors:** Wenzheng Zhang, Zhaorui Feng, Yitong Liu, Ruikang Zhong, Siyi Chen, Zexing Li, Bingyan Li, Dianna Liu, Lei Gao, Kaiwen Hu

**Affiliations:** 1 Beijing University of Chinese Medicine, Beijing, China; 2 Dongfang Hospital, Beijing University of Chinese Medicine, Beijing, China

**Keywords:** clinical decision support, generative artificial intelligence, large language models, lung cancer, multimodal foundation models, precision oncology

## Abstract

Generative artificial intelligence (GAI), particularly large language models (LLMs) and multimodal foundation models, represents a new generation of artificial intelligence technologies with emerging applications in healthcare. In lung cancer care, where clinical decisions increasingly require integration of imaging, pathology, molecular alterations, and rapidly evolving therapeutic evidence, GAI provides new opportunities to enhance clinical information synthesis and decision support. Recent studies have explored the application of GAI across the lung cancer care continuum, including screening and early detection, diagnosis and characterization, treatment decision-making, prognosis and disease monitoring, patient–clinician communication, and clinical workflow optimization. For example, multimodal foundation models such as M3FM have demonstrated the feasibility of jointly learning imaging and clinical information to support multiple lung cancer-related tasks within a unified architecture. In addition, oncology-specific LLMs trained on real-world clinical data have shown promise in predicting lung cancer progression by integrating longitudinal radiological and clinical information. However, most current applications remain at an exploratory or early validation stage, with substantial heterogeneity in model architectures, evaluation frameworks, and levels of clinical validation. Challenges related to evidence quality, data integration, safety, transparency, and regulatory oversight must be addressed before widespread clinical implementation. In this review, we provide a clinically oriented overview of the current applications of GAI across the lung cancer care continuum and discuss emerging developments, limitations, and future directions, including domain-specific models, multimodal systems, guideline-integrated decision support, and prospective validation frameworks.

## Introduction

Lung cancer remains the leading cause of cancer-related mortality worldwide, accounting for a substantial proportion of global cancer incidence and deaths (1). Advances in low-dose computed tomography (LDCT) screening, molecular diagnostics, targeted therapies, and immune checkpoint inhibitors have improved outcomes for selected patients with lung cancer. However, the overall prognosis remains poor, particularly among patients with advanced-stage disease (2, 3). Contemporary lung cancer care has become increasingly complex, requiring clinicians to integrate diverse sources of clinical information to support individualized treatment decisions (4, 5). This multidimensional decision-making process presents substantial challenges for clinicians and healthcare systems.

Artificial intelligence (AI) has emerged as a promising approach to addressing these challenges. Conventional AI systems, which are primarily based on discriminative models, have demonstrated value in specific tasks such as medical image analysis and risk prediction (6–8). More recently, generative artificial intelligence (GAI), particularly large language models (LLMs) and multimodal foundation models, has expanded the capabilities of AI by enabling natural language interaction, multimodal information integration, and advanced information synthesis. These advances have created new opportunities to support clinical decision-making, patient communication, and healthcare workflow optimization throughout the lung cancer care continuum (9, 10).

Despite the rapid expansion of GAI technologies, their application in lung cancer medicine remains incompletely characterized. Existing studies have primarily investigated GAI in isolated clinical scenarios, with substantial heterogeneity in study design, application domains, and evaluation methods. Comprehensive assessments of GAI across the lung cancer care continuum remain limited. Moreover, although numerous reviews have discussed AI applications in oncology, few have specifically synthesized the emerging evidence for GAI in lung cancer. These reviews have not comprehensively evaluated its clinical readiness, limitations, safety considerations, and future translational potential. Consequently, important questions remain regarding the level of clinical evidence supporting different GAI applications, the distinction between established and exploratory uses, and the challenges that must be addressed before safe and widespread implementation in lung cancer care.

In this narrative review, we provide a comprehensive and clinically oriented overview of the current applications of GAI across the lung cancer care continuum, including screening, diagnosis, molecular profiling, treatment decision-making, patient communication, and follow-up care. We also critically discuss the current evidence, implementation challenges, governance considerations, and future directions for the clinical integration of GAI in lung cancer care. By synthesizing the rapidly evolving literature, this review aims to provide clinicians, researchers, and policymakers with an evidence-based perspective on the opportunities and limitations of GAI in advancing precision lung cancer care.

## Methods

This article was conducted as a narrative review to summarize the current evidence on the applications, challenges, and future directions of GAI in lung cancer care. Given the rapidly evolving nature of this field and the substantial heterogeneity in study designs, AI models, and reported outcomes, a narrative review was considered the most appropriate approach to provide a clinically oriented synthesis of the available evidence.

A comprehensive literature search was performed in PubMed, Web of Science, and Embase from database inception to March 31, 2026, with the final search conducted on March 31, 2026. Only articles published in English were considered.

The search strategy combined controlled vocabulary (Medical Subject Headings [MeSH], where applicable) and free-text terms related to generative artificial intelligence and lung cancer. The PubMed search strategy was as follows:

((“Generative Artificial Intelligence”[Title/Abstract] OR “large language model*”[Title/Abstract] OR LLM*[Title/Abstract] OR “foundation model*”[Title/Abstract] OR “multimodal model*”[Title/Abstract] OR ChatGPT[Title/Abstract] OR GPT-4[Title/Abstract] OR GPT-5[Title/Abstract] OR Gemini[Title/Abstract] OR Claude[Title/Abstract] OR “Med-PaLM”[Title/Abstract]) AND (“lung cancer”[Title/Abstract] OR “lung neoplasm*”[Title/Abstract] OR “Lung Neoplasms”[Mesh] OR NSCLC[Title/Abstract] OR SCLC[Title/Abstract] OR “thoracic oncology”[Title/Abstract]))**.

Equivalent search strategies were adapted for Web of Science and Embase using database-specific subject headings and indexing terms.

Eligible publications included original research articles, review articles, consensus statements, and clinical guidelines addressing the application of GAI in lung cancer, including diagnosis, clinical decision support, patient communication, and healthcare workflow optimization. Studies focusing exclusively on non-medical AI applications or lacking clinical relevance were excluded.

**Figure 1:**
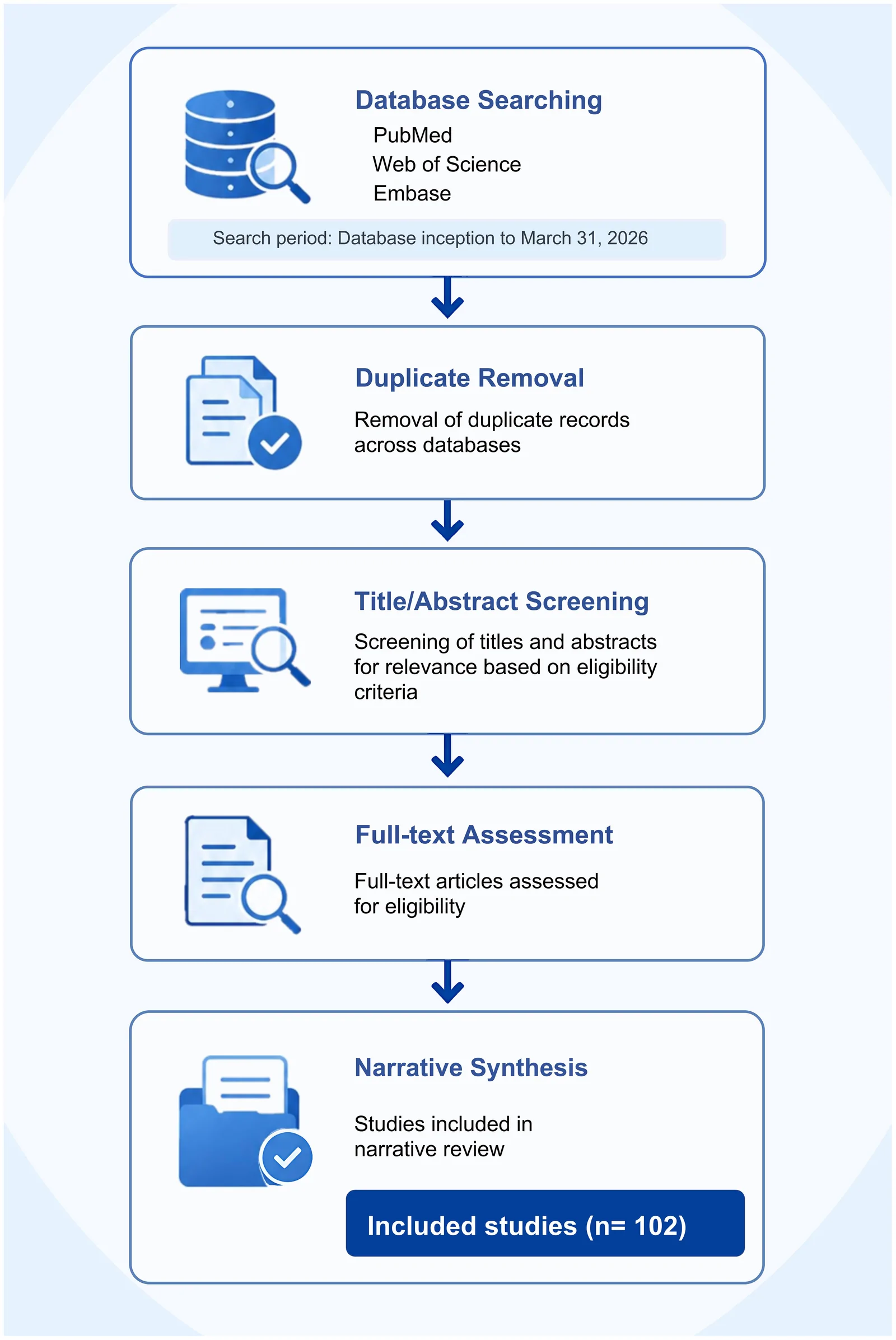
Literature search and study selection process for the narrative review. This flowchart illustrates the procedures used to identify, screen, assess, and include eligible studies investigating applications of generative artificial intelligence in lung cancer care. Literature searches were performed in PubMed, Web of Science, and Embase from database inception to March 31, 2026. Retrieved records were first screened for duplicate removal across databases. Subsequently, titles and abstracts were reviewed according to predefined eligibility criteria, followed by full-text assessment of potentially relevant articles. Studies were included based on their relevance to GAI applications in lung cancer diagnosis, treatment decision-making, prognosis, clinical workflow optimization, and future implementation. Finally, 102 studies were selected for narrative synthesis. Source: This figure was created by the authors and does not contain previously published material requiring permission.

Titles and abstracts were initially screened to identify potentially relevant studies, followed by full-text review where necessary. Potentially eligible articles subsequently underwent full-text assessment for final inclusion. For transparency of the literature identification and selection process, a simplified workflow is provided in **Figure 1**. After screening and eligibility assessment, 102 publications were included in the final narrative synthesis.

## GAI: technical foundations for lung cancer care

GAI refers to a class of AI systems that learn patterns from large-scale training datasets to produce novel synthetic outputs, including text, images, structured information, and multimodal content. GAI differs from conventional predictive AI by leveraging learned representations to generate, summarize, transform, and synthesize complex information rather than being limited to predefined prediction or classification tasks (9–18). In lung cancer care, modern GAI applications primarily rely on LLMs, which specialize in natural language understanding and generation, and multimodal foundation models, which integrate heterogeneous data modalities such as medical imaging, pathology, genomics, and electronic health records (16–22). By enabling language generation, multimodal information integration, and context-aware reasoning, GAI provides a unified framework to support diverse clinical tasks across the lung cancer care continuum (9, 10).

Many modern GAI systems, particularly large language models and multimodal foundation models, are based on transformer-based architectures and typically undergo large-scale pretraining followed by task-specific or domain-specific adaptation (23–25). LLMs have demonstrated potential in language-intensive applications, including clinical documentation, medical question answering, literature summarization, and guideline interpretation (26–35). Multimodal foundation models further extend these capabilities by integrating complex clinical data sources (20, 27–29). Alignment strategies such as reinforcement learning with human feedback (RLHF) have been introduced to improve instruction following, safety, and the alignment of model outputs with human preferences. These improvements may facilitate their application in clinical settings (36). These advances provide the technical foundation for the expanding applications of GAI in lung cancer care discussed in the following sections.

## Clinical applications of GAI across the lung cancer care continuum

**Figure 2:**
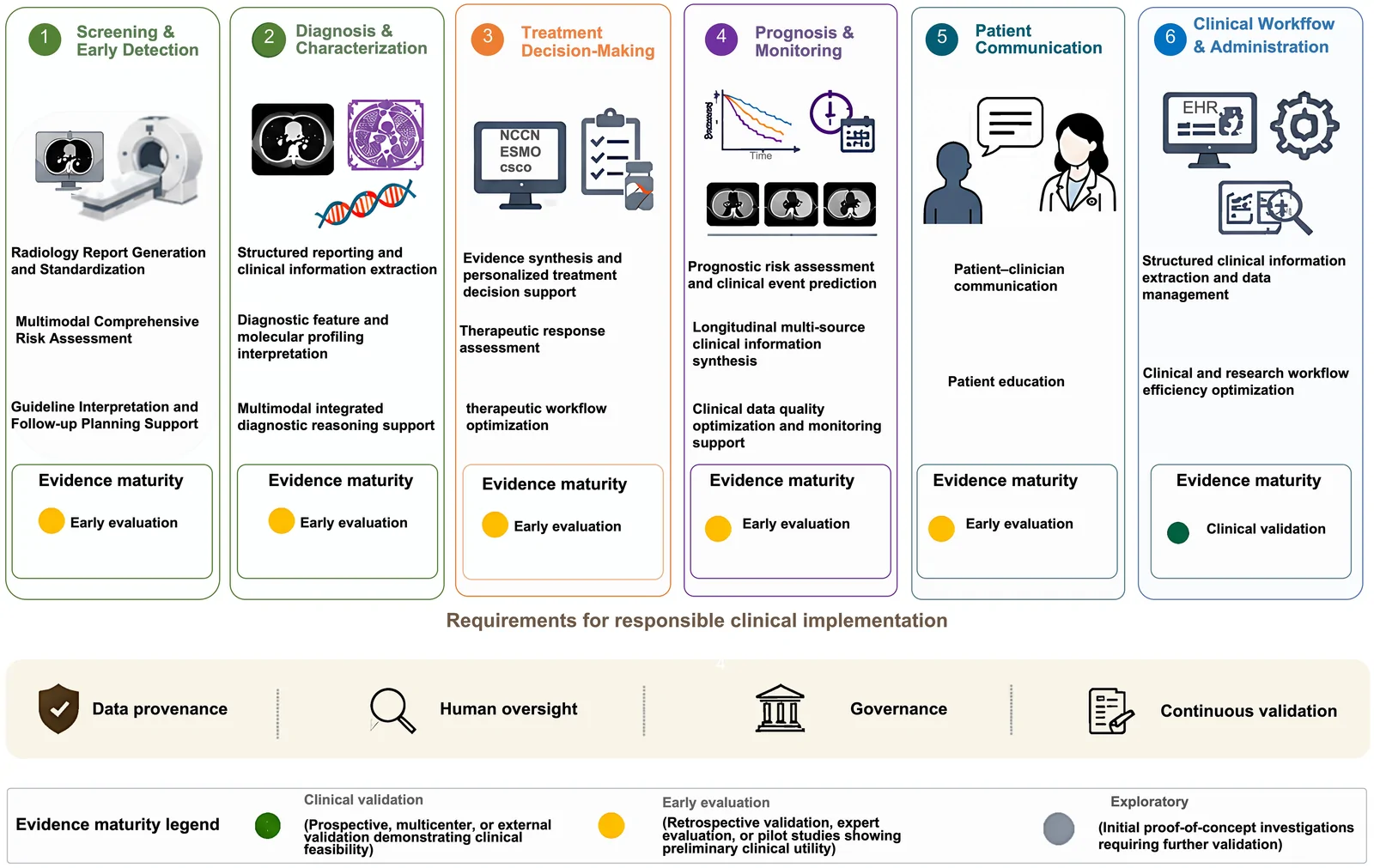
Clinical applications of GAl across the lung cancer care continuum. This figure summarizes the current applications and translational potential of GAI across six major domains of lung cancer care, including screening and early detection, diagnosis and characterization, treatment decision-making, prognosis and monitoring, patient communication, and clinical workflow optimization. GAI applications are presented according to their clinical roles, ranging from multimodal information synthesis and evidence support to communication enhancement and workflow assistance. The evidence maturity indicators distinguish applications with prospective or external clinical validation from those supported mainly by retrospective studies, pilot evaluations, or exploratory proof-of-concept investigations. The figure highlights that most current GAI applications remain in early evaluation stages and should be implemented as assistive technologies rather than autonomous decision-makers. Responsible clinical implementation requires foundational elements including reliable data provenance, human oversight, governance frameworks, and continuous validation to ensure safety, transparency, and clinical effectiveness. GAI, generative artificial intelligence; EHR, electronic health record; NCCN, National Comprehensive Cancer Network; ESMO, European Society for Medical Oncology; CSCO, Chinese Society of Clinical Oncology. Source: This figure was created by the authors and does not contain previously published material requiring permission.

Building upon the technical foundations described above, GAI has been increasingly explored across the lung cancer care continuum, including screening and early detection, diagnosis and characterization, treatment decision-making, prognosis and disease monitoring, and cross-cutting clinical support functions such as patient communication and workflow optimization. **Figure 2** provides an overview of these applications and their current evidence maturity, while also highlighting the key requirements for responsible clinical implementation. Most applications remain in early evaluation, and only a limited number have reached clinical validation. **Table 1** summarizes representative studies and their supporting evidence across these clinical domains. The following sections examine the current evidence, clinical value, and remaining limitations of GAI in each stage of lung cancer care.

**Table 1:** Summary of current evidence and clinical readiness of GAI applications in lung cancer care.

Clinical application	Examples of GAI	Key studies	Clinical contexts and populations	Validation type	Evaluation outcomes	Evidence status	Clinical implementation readiness
Screening and Early Detection	Multimodal foundation models (M3FM)	Developed M3FM for multitask lung cancer screening using CT imaging and clinical data (47).	LDCT lung cancer screening populations	Retrospective internal validation	Improved performance in multiple screening tasks and lung cancer risk prediction	Validation study	Early-stage; requires external and prospective validation
	LLMs (GPT-3.5, GPT-4, Claude3, GPT-4o, DeepSeek-R1)	Multicenter evaluation of LLM-generated management recommendations from LDCT reports (49).	Lung cancer screening; 148 anonymized LDCT reports from three institutions	Retrospective multicenter external validation	Demonstrated feasibility of LLM-generated screening recommendations	Validation study	Early-stage; requires prospective validation
Diagnosis and Characterization	LLMs (ChatGPT, GPT-4)	LLM-based extraction of oncologic phenotypes from free-text lung cancer CT reports (55)	Lung cancer diagnosis and follow-up; 424 CT reports from 424 patients	Retrospective single-center validation	Demonstrated accurate extraction of oncologic features and progression- related information from CT reports	Validation study	Early-stage; requires external and prospective validation
	Multimodal LLM (ChatGPT-4V)	Evaluation of ChatGPT-4V for chest CT interpretation and disease classification (61)	Chest CT diagnosis; 60 cases including NSCLC, COVID-19, and control cases from TCIA	Retrospective proof-of- concept evaluation with radiologist assessment	Demonstrated limited diagnostic performance of ChatGPT-4V for direct CT interpretation, with variable accuracy across disease categories.	Proof-of-concept study	Early-stage; requires further optimization and clinical validation
	LLM (ChatGPT-3.5) with prompt engineering	LLM-based extraction of structured information from lung cancer pathology reports (71)	>1,000 pathology reports evaluated against expert- curated structured data	Retrospective validation against expert annotations	Demonstrated feasibility of LLM-based pathology information extraction with 89% accuracy in lung cancer classification	Validation study	Early-stage; requires external validation and integration into clinical workflows
	Multimodal LLM (ChatGPT-4o)	Evaluation of ChatGPT-4o for lung tumor histopathological image-based diagnosis (77)	250 lung tumor HE-stained images including LUAD, LUSC, and other lung lesions	Retrospective evaluation against expert pathologists	Showed rapid pathology image interpretation potential but lower accuracy than experts	Proof-of-concept study	Early-stage; may support pathologist decision-making but requires external validation and workflow integration
Treatment Decision- Making	LLMs (Gemini 2.5 Pro, ChatGPT- 4o) with task- specific prompting	LLM-based automated immunotherapy response assessment from lung cancer PET/CT reports (86)	Lung cancer immunother-apy monitoring; 36 patients with pre- and post- treatment PET/CT reports	Retrospective evaluation against expert nuclear medicine specialist assessment	Demonstrated high agreement with expert assessment for PET/CT-based immunotherapy response classification using EORTC criteria	Proof-of-concept study	Early-stage; requires larger multicenter validation and integration into clinical workflows
	General- purpose LLMs (ChatGPT, DeepSeek, Gemini)	Evaluation of LLMs for evidence synthesis and clinical reasoning in advanced NSCLC immunotherapy (79)	Advanced NSCLC; PD-1/PD-L1 inhibitor treatment evidence evaluation	Comparative benchmark evaluation using expert-based assessment	Demonstrated potential for treatment-related evidence synthesis and clinical reasoning, but showed limitations in source verification and methodological rigor	Proof-of-concept evaluation	Early-stage; requires evidence grounding and clinical oversight
	Generative AI system (GenDSA-V2) for low-dose DSA imaging	Prospective validation of GenDSA-V2 for radiation reduction in DSA-guided procedures including lung cancer interventions (88)	417 lung cancer patients undergoing DSA-guided procedures	Prospective multicenter clinical validation	Reduced radiation exposure by approximately two-thirds while maintaining procedural efficiency and safety	Prospective validation	Demonstrated clinical feasibility; requires broader implementation evaluation
Prognosis and Monitoring	Hybrid machine learning and LLM pipeline	LLM-augmented prediction of breakthrough pain episodes in hospitalized lung cancer patients (90)	Inpatient lung cancer care; 266 hospitalized patients requiring pain management	Retrospective cohort evaluation using electronic health record data	Improved prediction of breakthrough pain episodes through multimodal EHR integration	Proof-of-concept study	Demonstrated predictive feasibility; requires prospective validation and workflow integration
	Oncology- specific LLM (Woollie)	Predicting cancer progression using longitudinal clinical information and radiology reports in patients with lung cancer and other malignancies (89)	Multicancer cohort including lung cancer patients; 39,319 radiology impression notes from 4,002 patients	Retrospective cohort study with external validation using independent institutional data	Achieved an AUROC of 0.97 for cancer progression prediction in the MSK cohort and 0.88 in external validation; lung cancer detection achieved an AUROC of 0.95 in the UCSF cohort	Proof-of-concept study with external validation	Demonstrated predictive feasibility; requires prospective validation and integration into longitudinal lung cancer monitoring workflows
Patient Communication	LLM (ChatGPT)	Evaluation of ChatGPT responses to lung cancer surgery questions (97)	Patient education; 30 surgery- related questions assessed by thoracic surgeons	Expert evaluation of ChatGPT- generated responses	High-quality responses but frequent inaccuracies and limited surgical specificity	Proof-of-concept study	Potential for patient communication support; requires oversight
Clinical Workflow and Administration	Fine-tuned LLM pipeline for clinical data extraction	Multicenter validation of LLM-assisted real-world data extraction in lung cancer cohorts (100)	Multicenter lung cancer cohort; 10,327 patients from 10 centers	Multicenter validation against manual abstraction with expert adjudication	Reduced extraction error rates and improved consistency compared with manual abstraction; hybrid AI-human review further improved accuracy	Multicenter validation study	Demonstrated feasibility for scalable clinical data extraction; requires integration into institutional workflows

This table summarizes representative GAI technologies, key clinical applications, study populations, validation approaches, evaluation outcomes, evidence status, and potential readiness for clinical implementation across different stages of lung cancer management.

### Screening and early detection

Lung cancer screening with LDCT has become an established strategy for reducing lung cancer mortality among high-risk populations (3, 37). With the widespread implementation of LDCT screening programs, the number of detected pulmonary nodules has increased substantially, generating a need for standardized reporting, guideline-based follow-up, patient counseling, and longitudinal management (38). Conventional artificial intelligence has already demonstrated considerable value in this setting by improving pulmonary nodule detection, segmentation, characterization, and malignancy prediction through image-based analysis (39–42). In contrast, the current role of GAI is not to replace image interpretation, but to enhance the downstream clinical workflow that follows radiological assessment.

One important application of GAI is the generation and standardization of radiology reports (43). LLMs can automatically summarize imaging findings, generate structured reports, standardize terminology according to reporting systems such as Lung-RADS, and produce concise clinical summaries for referring physicians. These capabilities have the potential to improve reporting consistency, reduce documentation burden, and facilitate communication among radiologists, pulmonologists, thoracic surgeons, and primary care physicians (44–46).

Recent advances in multimodal generative AI have further expanded these capabilities. Rather than relying solely on textual information, multimodal foundation models jointly process imaging and clinical data to support multiple screening-related tasks. For example, the Medical Multimodal Multitask Foundation Model (M3FM) was developed using large-scale multimodal and multitask learning to integrate chest CT images with clinical information across 17 lung cancer screening-related tasks. M3FM improved lung cancer risk prediction performance by up to 20% compared with state-of-the-art models, highlighting the potential of multimodal GAI models for comprehensive risk assessment and integrated screening applications (47). These findings illustrate the emerging potential of multimodal GAI to complement conventional imaging AI throughout the lung cancer screening pathway.

GAI also shows promise in guideline interpretation and follow-up planning. By integrating imaging findings with current screening guidelines, GAI systems may assist clinicians in generating draft follow-up recommendations and explaining surveillance strategies, while final management decisions remain under physician supervision (48–50).

Nevertheless, current evidence supporting GAI in lung cancer screening remains limited. Although some studies have demonstrated feasibility through retrospective validation, many remain at an early evaluation stage, with limited prospective assessment and real-world implementation evidence across diverse healthcare systems. Importantly, a study evaluating GPT-3.5 and GPT-4 for Fleischner Society guideline-based pulmonary nodule management demonstrated limited performance, particularly in complex clinical reasoning and biopsy recommendations. These findings highlight the need for continued clinician oversight when applying GAI to guideline-driven screening decisions (48). Recent comparative evaluations of multiple state-of-the-art LLMs further demonstrated that several models performed well in answering questions related to lung cancer screening and pulmonary nodule management. However, incorrect or inconsistent recommendations remained common, indicating that these systems should not yet be relied upon as independent clinical decision-makers (50).

Overall, current evidence suggests that GAI should be viewed as a complementary technology that augments, rather than replaces, conventional imaging AI in lung cancer screening. Predictive AI remains essential for nodule detection and malignancy assessment, whereas GAI may improve reporting, information synthesis, and guideline-based clinical support. Future prospective studies are needed to define its clinical value while ensuring appropriate human oversight.

### Diagnosis and characterization

Accurate diagnosis and comprehensive characterization of lung cancer require the integration of multiple data sources, including imaging, histopathology, molecular profiling, and other clinically relevant patient characteristics (4, 5). While conventional artificial intelligence has achieved impressive performance in individual diagnostic tasks such as pulmonary nodule detection and image classification, these systems are generally optimized for single-modality analysis (11, 12). In contrast, GAI is increasingly being explored as a complementary technology capable of integrating heterogeneous diagnostic information, generating clinically interpretable summaries, and supporting diagnostic reasoning throughout the lung cancer diagnostic pathway (10).

#### Imaging

Although conventional AI has substantially improved pulmonary nodule detection, segmentation, radiomic analysis, and malignancy prediction (51, 52), recent advances in GAI have shifted attention toward supporting downstream radiology workflows rather than replacing image interpretation itself (42). Current GAI applications primarily aim to transform imaging findings into structured, clinically actionable knowledge that facilitates diagnosis, staging, and longitudinal management throughout the lung cancer care pathway (43, 53).

One of the most mature applications of GAI is the extraction and structuring of information from free-text radiology reports (54–58). Recent studies have demonstrated that LLMs can automatically extract clinically relevant imaging features, including tumor location, lesion size, lymph node involvement, distant metastases, TNM-related findings, and actionable incidental findings, from unstructured CT or PET/CT reports and convert them into standardized structured data without requiring task-specific model development (56–58). These capabilities may facilitate clinical documentation, longitudinal follow-up, registry development, and large-scale clinical research while reducing the manual burden associated with radiology data abstraction.

Beyond information extraction, GAI has demonstrated considerable potential for automated radiology report generation and summarization. LLMs can generate concise and standardized summaries of chest CT and PET/CT reports, while multimodal generative models integrate imaging features with clinical information to draft comprehensive radiology reports. More recently, multimodal generative frameworks have demonstrated encouraging performance in automatically generating TNM-aware whole-body PET/CT reports for patients with lung cancer, showing good cross-center generalizability and high physician-rated report quality (59, 60). These advances suggest that GAI may improve reporting consistency, reduce documentation burden, and enhance communication among radiologists, pulmonologists, thoracic surgeons, and multidisciplinary teams.

Recent developments have further extended the role of GAI from report generation toward imaging-based clinical reasoning. Multimodal GAI models have begun to explore direct interpretation of medical images. For example, ChatGPT-4V was evaluated for direct chest CT interpretation and disease classification in a proof-of-concept study involving 60 cases, including NSCLC, COVID-19, and control cases. The model achieved an overall diagnostic accuracy of 56.76%; for NSCLC detection, it showed a sensitivity of 27.27% and a specificity of 60.47%, suggesting limited performance in direct CT-based lung cancer interpretation (61). Other approaches have focused on integrating radiology reports with clinical guidelines and evidence-based knowledge through methods such as RAG, knowledge-enhanced prompting, or multi-agent frameworks. These systems have shown potential to support lung cancer–related tasks, including TNM staging, imaging interpretation, and downstream clinical decision-making (55, 62–64). By combining imaging information with clinical context and medical knowledge, GAI may generate clinically meaningful recommendations to assist radiologists and multidisciplinary teams during diagnostic evaluation and follow-up.

Despite these encouraging advances, current evidence remains preliminary, and GAI should currently be regarded as an assistive technology rather than an autonomous imaging diagnostic system. Most published studies are based on retrospective evaluations, proof-of-concept investigations, or early validation studies. Prospective evaluation and real-world implementation across diverse clinical settings remain limited. Evaluations of multimodal LLMs have shown promising capabilities in report interpretation and clinical reasoning. However, their diagnostic accuracy remains inferior to expert radiologists in complex imaging tasks, and challenges related to hallucinations, staging consistency, and generalizability persist (63, 65, 66). Consequently, GAI is more appropriately viewed as a complement to radiologists and conventional imaging AI systems, with its greatest current value lying in structured reporting, evidence synthesis, and workflow optimization rather than primary image interpretation.

#### Pathology

Histopathological examination remains the gold standard for lung cancer diagnosis and provides essential information for tumor classification, grading, and therapeutic decision-making (4, 5). While conventional computational pathology has achieved remarkable success in tumor detection, segmentation, and histological classification (67–70), the emerging role of GAI lies primarily in transforming pathological information into structured, clinically interpretable knowledge rather than replacing microscopic image interpretation.

One of the most mature applications of GAI is the extraction and structuring of information from free-text pathology reports. Recent studies have demonstrated that LLMs can accurately extract clinically relevant pathological findings, including histological subtype, tumor differentiation, lymphovascular invasion, margin status, TNM-related features, and biomarker results, from unstructured pathology reports. These models can further convert pathology narratives into standardized datasets for clinical research and data analysis (71, 72). Lung cancer-specific LLMs have further demonstrated superior performance in generating structured pathological databases and standardized pathology reports compared with general-purpose LLMs and non-specialist clinicians (73). These capabilities may improve documentation efficiency and facilitate clinical research, registry construction, and real-world data analysis.

Beyond structured information extraction, GAI has shown considerable promise in pathology report generation and summarization. Recent multimodal large language models can integrate whole-slide images with pathological findings to generate comprehensive pathology reports. These models have also shown potential for maintaining reporting consistency across institutions (73, 74). By combining visual and textual information, these systems may reduce reporting variability, improve documentation quality, and facilitate communication between pathologists and multidisciplinary teams, thereby supporting more standardized pathological workflows.

Another emerging application is explainable pathological feature interpretation. Unlike conventional deep-learning models, which often function as “black boxes,” multimodal GAI models can identify diagnostically relevant pathological features. They can also provide natural-language explanations of their clinical significance (75–77). Recent studies in lung adenocarcinoma have demonstrated that GAI can assist in pathological grading and extract clinically relevant morphological features, supporting more accurate and interpretable pathological assessment (75). Furthermore, knowledge-enhanced multimodal frameworks that integrate pathological images with medical literature have shown encouraging results in improving histological subtype classification and pathological reasoning, suggesting that GAI may facilitate more transparent and clinically interpretable computational pathology (76).

Despite these encouraging advances, current evidence indicates that GAI should be regarded as an assistive tool rather than a replacement for pathologists. Most published studies remain retrospective or proof-of-concept investigations, and prospective multicenter validation is still limited. Although multimodal LLMs have demonstrated promising performance in pathological report generation and feature interpretation, their diagnostic accuracy for direct image-based classification remains inferior to that of experienced pathologists, particularly in challenging or uncommon cases (77, 78). Consequently, the greatest current value of GAI lies in pathology information extraction, structured reporting, explainable feature interpretation, and workflow optimization, while definitive pathological diagnosis continues to require expert review and human oversight.

#### Molecular profiling

Comprehensive molecular profiling has become a cornerstone of precision medicine in lung cancer. The identification of actionable biomarkers, including EGFR, ALK, ROS1, BRAF, KRAS G12C, MET exon 14 skipping, RET fusions, HER2 mutations, and PD-L1 expression, directly guides the selection of targeted therapies and immunotherapy (4, 5). These molecular findings increasingly influence clinical trial enrollment and treatment sequencing. As molecular testing becomes more comprehensive through next-generation sequencing (NGS), interpreting increasingly complex genomic reports and integrating rapidly evolving therapeutic evidence have become major challenges in routine clinical practice.

Rather than performing genomic analysis itself (11), GAI primarily supports the interpretation and contextualization of molecular testing results. Medically aligned large language models have shown potential to summarize NGS reports and explain the clinical significance of detected genomic alterations. They may also assist in identifying actionable alterations and linking molecular findings with approved targeted therapies, immunotherapy options, and relevant clinical guidelines (29, 79). By transforming lengthy molecular reports into concise, clinically interpretable summaries, GAI may facilitate communication between molecular pathologists, oncologists, and multidisciplinary tumor boards while reducing the cognitive burden associated with increasingly complex genomic information.

Although GAI systems can efficiently summarize molecular information and relevant evidence, their performance remains limited in complex clinical scenarios. Challenges include incomplete evidence integration, unreliable citation generation, and insufficient clinical reasoning when multiple therapeutic factors need to be considered (79). Recent evaluations of LLMs in clinical decision-making and evidence-based medicine have highlighted these limitations, emphasizing the need for clinician oversight and multidisciplinary evaluation in biomarker-driven treatment decisions (80, 81). Therefore, GAI should currently be regarded as an assistive tool to support, rather than replace, expert interpretation of molecular profiling results.

#### Multimodal clinical reasoning

The greatest long-term potential of generative AI may lie not in improving the performance of individual diagnostic modalities but in integrating heterogeneous clinical information into unified clinical reasoning. Lung cancer management routinely requires clinicians to synthesize findings from multiple sources, including thoracic CT or PET/CT, histopathology, molecular profiling, laboratory investigations, and electronic health records. These data must be interpreted alongside continuously evolving clinical guidelines to support diagnosis and treatment decisions (4, 5). This complex reasoning process extends beyond the capabilities of conventional task-specific AI systems, which are typically designed to analyze a single data modality.

Recent advances in multimodal large language models and foundation models have begun to address this challenge by combining imaging, pathology, molecular, and clinical information within a unified framework. Early studies have demonstrated promising applications in automated TNM staging from radiology reports, multimodal PET/CT report generation, structured extraction of clinically relevant findings, and integrated diagnostic support across multiple data sources (59, 74). Multimodal foundation models such as M3FM have shown the feasibility of jointly learning imaging and clinical information to support multiple lung cancer-related tasks within a single architecture (47). Emerging vision-language systems have also demonstrated the ability to generate clinically coherent interpretations by integrating radiological and textual information in lung cancer care (60). These developments suggest that multimodal GAI may evolve from assisting isolated diagnostic tasks toward supporting comprehensive clinical assessment across the lung cancer care pathway.

Nevertheless, this field remains at an early stage of clinical translation. Most published studies are retrospective or proof-of-concept investigations, and robust prospective validation across diverse institutions remains limited. Important challenges, including multimodal data harmonization, interoperability between healthcare systems, explainability, privacy protection, and regulatory oversight, must be addressed before these technologies can be routinely incorporated into clinical practice. Accordingly, current multimodal GAI systems should be regarded as decision-support tools that complement, rather than replace, multidisciplinary clinical expertise.

### Treatment decision-making

Treatment decision-making in lung cancer has become increasingly complex with the rapid evolution of precision oncology and expanding therapeutic options. Contemporary management encompasses surgery, radiotherapy, chemotherapy, targeted therapy, immunotherapy, and multimodal combination strategies. These decisions require integration of tumor stage, histopathological subtype, molecular alterations, PD-L1 expression, patient characteristics, previous treatment history, and continuously evolving clinical evidence to develop individualized treatment plans (4, 5). This complexity places substantial demands on clinicians in ensuring that therapeutic decisions remain both evidence-based and personalized.

One of the most promising applications of GAI in this setting is evidence synthesis and guideline interpretation. LLMs can rapidly summarize recommendations from major oncology guidelines, integrate findings from recent clinical trials, and generate concise evidence summaries for specific clinical scenarios (13, 19, 80). Such capabilities may help clinicians efficiently navigate frequently updated recommendations from organizations such as NCCN, ESMO, and CSCO, thereby reducing the time required for evidence retrieval and supporting more consistent evidence-based practice. Nevertheless, current evidence indicates that LLMs remain limited in complex clinical reasoning. Although they can generate clinically relevant summaries, their ability to integrate complex evidence, critically appraise supporting literature, and make nuanced therapeutic judgments remains insufficient (82, 83). Comparative evaluations have demonstrated deficiencies in citation reliability, evidence integration, and clinical reasoning, suggesting that these systems should serve as assistive tools rather than independent decision makers (48, 50, 83).

Beyond evidence synthesis, GAI has considerable potential to facilitate personalized treatment planning in precision oncology. Rather than directly identifying genomic alterations, GAI may assist clinicians by summarizing molecular reports, contextualizing actionable biomarkers, and linking genomic findings with relevant therapeutic evidence and clinical guidelines (84, 85). In NSCLC, these applications may support the interpretation of clinically important biomarkers, including EGFR, ALK, ROS1, KRAS G12C, MET exon 14 skipping, RET fusions, HER2 mutations, and PD-L1 expression. By integrating molecular information with available treatment options and emerging clinical evidence, GAI may help improve the efficiency of precision oncology workflows.

An emerging application is treatment response assessment during longitudinal follow-up. Recent evidence suggests that GPT-4o and Gemini can automatically analyze serial PET/CT reports and classify immunotherapy responses according to the European Organization for Research and Treatment of Cancer (EORTC) criteria (86). In a study involving 36 lung cancer patients, Gemini 2.5 Pro achieved an overall accuracy of 94% and showed strong agreement with expert assessment (Cohen’s κ = 0.907), while ChatGPT-4o achieved 100% accuracy with perfect agreement (Cohen’s κ = 1.000). By summarizing interval imaging changes and generating standardized response assessments, these systems may assist clinicians in monitoring therapeutic efficacy and documenting longitudinal disease changes. Although these early results are encouraging, prospective validation across larger and more diverse patient populations remains necessary before routine clinical implementation.

Beyond individual decision support, GAI may also play a role in enhancing multidisciplinary team (MDT) discussions (87). By automatically summarizing lung cancer patient histories and consolidating radiological, pathological, and molecular findings, GAI can facilitate more structured MDT discussions. It can also retrieve relevant guideline recommendations and supporting evidence, reducing the time required for case preparation.

Beyond cognitive decision support, GAI is beginning to expand into image-guided therapeutic procedures for lung cancer. A recent randomized controlled trial demonstrated that generative AI-based reconstruction of low-dose digital subtraction angiography (DSA) significantly reduced radiation exposure, while maintaining procedural efficacy and safety, in patients undergoing DSA-guided interventions, including those with lung cancer (88). Although further validation in lung cancer–specific interventional settings is warranted, these findings highlight the emerging potential of GAI to improve procedural imaging quality, optimize interventional workflows, and enhance the safety of image-guided cancer treatment.

Overall, while GAI holds considerable promise in augmenting treatment decision-making in lung cancer, its safe and effective clinical implementation will depend on rigorous validation, continuous updating, and seamless integration into clinician-led workflows.

### Prognosis and disease monitoring

Accurate prognostic assessment and longitudinal disease monitoring are critical for optimizing lung cancer management. These tasks require continuous integration of imaging findings, treatment history, pathological and molecular information, laboratory results, and clinical symptoms across the disease trajectory (4, 5). GAI is not intended to replace established prognostic models but may serve as an assistive tool for organizing and interpreting longitudinal clinical information. By synthesizing heterogeneous records, GAI may facilitate structured follow-up summaries, recognition of clinically meaningful changes, and more efficient multidisciplinary evaluation.

Recent studies have demonstrated the potential of GAI in lung cancer surveillance and risk assessment. In external validation, the model achieved an AUROC of 0.95 for lung cancer detection (89). These findings highlight the potential of oncology-specific LLMs to improve risk assessment and support longitudinal lung cancer management through integration of clinical information. A hybrid model incorporating machine learning and LLMs was developed to predict breakthrough cancer pain episodes in hospitalized lung cancer patients using longitudinal electronic health record data. By integrating structured clinical variables with unstructured clinical notes, this approach achieved accuracies of 0.876 and 0.917 for 48-hour and 72-hour prediction, respectively, with improved sensitivity after LLM augmentation, suggesting its potential to support proactive symptom management during lung cancer care (90). Beyond prediction tasks, GAI can also improve the quality of longitudinal clinical data. For example, LLM-based extraction combined with longitudinal smoothing strategies has been explored to improve the quality and consistency of smoking history documentation from clinical notes. LLMs achieved over 96% accuracy across multiple smoking-related variables, with external validation demonstrating robust performance (97.5–98.8% accuracy). These findings suggest that such approaches may provide more reliable longitudinal data to support lung cancer surveillance and risk assessment (91).

Despite these promising applications, current evidence remains preliminary. Most studies remain retrospective or early-stage validation studies, with limited prospective and multicenter evaluation. Although GAI has demonstrated potential in integrating longitudinal clinical information, supporting risk assessment, and predicting selected clinical events, evidence of clinical benefit remains limited. Robust evidence demonstrating improvements in long-term outcomes, or routine follow-up management is still lacking. Therefore, GAI should currently be regarded as an assistive tool that augments clinician-led disease monitoring rather than an autonomous system for clinical decision-making. Future studies should prioritize prospective validation, multimodal integration, and evaluation of real-world clinical impact.

### Cross-cutting clinical support

Beyond stage-specific applications, GAI provides several cross-cutting capabilities that support the entire lung cancer care continuum. It has the potential to improve both patient–clinician communication and patient education. By translating complex clinical information into accessible language, GAI can explain pulmonary nodule findings, molecular testing results, PET/CT reports, treatment options, and follow-up recommendations. These capabilities may improve health literacy, strengthen patient engagement, and support shared decision-making (92–100).

In addition to communication support, GAI may improve clinical workflow efficiency by facilitating the organization and extraction of clinical information. Lung cancer care generates large volumes of unstructured medical data, including clinical notes, treatment records, and follow-up documentation. Recent studies have shown that LLM-based approaches can accurately extract structured clinical variables from real-world oncology records and reduce the workload associated with manual data abstraction. In lung cancer cohorts, AI-assisted extraction achieved lower error rates and greater consistency than manual abstraction, while hybrid AI–human workflows further improved data quality (100). These applications may enhance clinical information management and support more efficient research and clinical workflows. However, prospective evaluations are still needed to determine their impact on routine clinical practice and patient outcomes.

Collectively, these cross-cutting capabilities complement the stage-specific applications discussed above. They highlight the principal role of GAI in lung cancer care as an integrative tool for organizing information, supporting communication, and enhancing clinical efficiency, rather than replacing specialist expertise.

## Challenges and limitations

### Hallucination and reliability

One of the most critical limitations of GAI in clinical medicine is the phenomenon of “hallucination,” whereby models generate information that is plausible in appearance but factually incorrect, incomplete, or entirely fabricated. Unlike traditional rule-based systems, GAI models produce outputs based on learned statistical patterns rather than verified knowledge sources, which increases the risk of generating misleading or non-evidence-based content (101–103).

In lung cancer care, hallucinations may manifest as incorrect characterization of pulmonary nodules, inaccurate interpretation of TNM stage, erroneous summarization of pathology or molecular reports (e.g., EGFR, ALK, ROS1, or PD-L1 status), or treatment recommendations that are inconsistent with current NCCN, ESMO, or CSCO guidelines. Such errors may not always be readily identifiable, particularly when outputs are presented in fluent and authoritative language. This creates a risk of over-trust, where clinicians or patients may accept AI-generated information without sufficient critical evaluation (104, 105).

Reliability is further challenged by the variability of model performance across different clinical scenarios. Recent studies suggest that LLMs may perform well in information organization and structured summarization, but their reliability decreases when tasks require complex clinical reasoning, guideline interpretation, or individualized decision-making. For example, evaluations based on the Fleischner Society guidelines showed that GPT-3.5 and GPT-4 exhibited suboptimal performance in pulmonary nodule management, particularly for biopsy recommendations and complex clinical reasoning (48). Recent assessments of lung cancer-related question answering also reported inconsistent responses across clinically challenging scenarios (66). Collectively, these findings indicate that current GAI systems are substantially more reliable for knowledge organization than for autonomous clinical reasoning.

These limitations have important implications for patient safety. In lung cancer practice, inaccurate outputs may result in inappropriate pulmonary nodule surveillance recommendations, delayed identification of actionable molecular alterations, incorrect staging, or suboptimal treatment selection. These errors may ultimately affect downstream clinical management. Consequently, GAI should be regarded as an assistive technology rather than an independent clinical decision-maker, and all AI-generated outputs require careful review by qualified clinicians.

To mitigate these risks, continuous human oversight remains essential. Clinicians should critically evaluate AI-generated content against established evidence and contemporary clinical guidelines. At the same time, ongoing efforts are focused on improving model alignment, uncertainty estimation, and error detection through domain-specific optimization. Emerging evidence also suggests that RAG can substantially reduce hallucinations by grounding model responses in authoritative external knowledge sources rather than relying solely on parametric memory. In lung cancer, RAG-enhanced systems have already demonstrated improved performance in tasks such as TNM staging by incorporating guideline-based knowledge into the inference process (64). Future integration of continuously updated lung cancer–specific knowledge bases with RAG and RLHF may further enhance factual accuracy and reduce hallucinations. Such knowledge bases may incorporate NCCN, ESMO, and CSCO guidelines, molecular oncology databases, and multidisciplinary treatment protocols to improve the clinical reliability of GAI in lung cancer care.

### Data bias and fairness

Bias in training data represents a significant challenge for the safe and equitable deployment of GAI in lung cancer care. Lung cancer is a highly heterogeneous disease involving diverse demographic characteristics, environmental exposures, molecular subtypes, and treatment patterns. Insufficient representation of specific patient populations may therefore compromise model generalizability and clinical reliability.

This issue is particularly relevant in precision oncology. The distribution of actionable genomic alterations varies substantially across populations; for example, EGFR mutations are more frequently observed in Asian patients with NSCLC than in many Western populations, whereas other molecular alterations, including KRAS, ALK, and ROS1 abnormalities, show different prevalence patterns across ethnic groups. Models developed predominantly from datasets lacking adequate demographic and molecular diversity may generate biased interpretations of biomarker profiles or therapeutic recommendations. Such biases may affect the equitable delivery of targeted therapies and immunotherapies (106–108).

Bias may also arise from the heterogeneous nature of clinical data used for GAI development. In thoracic imaging, variations in CT acquisition protocols, scanner platforms, reconstruction algorithms, and institutional reporting practices may influence model performance and limit cross-center applicability (109, 110). Similarly, pathology and molecular datasets may be affected by differences in diagnostic standards, sequencing platforms, and clinical workflows (111, 112). These challenges are particularly important for multimodal GAI systems, which require the integration of imaging, pathology, genomic, and clinical information from diverse healthcare environments.

Addressing these challenges requires deliberate efforts to improve data diversity and representativeness in model development. This includes incorporating datasets from diverse geographic regions, healthcare systems, and patient populations. In addition, standardized evaluation frameworks should include subgroup analyses to assess model performance across demographic and clinical categories, including molecularly defined lung cancer populations. Transparent reporting of dataset composition and rigorous external validation across diverse healthcare settings will be essential. These measures will help ensure that GAI systems provide equitable benefits across different lung cancer populations. Importantly, clinicians should remain aware of potential biases when interpreting GAI outputs and consider their applicability to individual patient contexts.

### Lack of interpretability

A fundamental challenge in the clinical application of GAI is the limited interpretability of model outputs (80). Traditional statistical models and rule-based clinical tools often allow decision pathways to be explicitly traced and justified. In contrast, large language and multimodal foundation models operate through highly complex and non-transparent internal representations. As a result, although GAI systems may generate clinically plausible responses, the rationale and evidence supporting these outputs are often difficult to fully elucidate.

In the context of lung cancer care, this lack of interpretability poses significant barriers to clinical adoption. Diagnostic and therapeutic decisions require transparent, evidence-based justification (112–117). For example, GAI-assisted interpretation of thoracic imaging, PET/CT assessment, or pathology findings should allow clinicians to understand which imaging features, pathological characteristics, or clinical information contributed to the generated conclusions. Similarly, when recommending biomarker-driven therapies for NSCLC, such as EGFR- or ALK-targeted treatments, GAI outputs should be linked to specific molecular alterations, clinical guidelines, and supporting evidence. Such connections are essential for improving transparency, traceability, and clinical reliability (116, 117).

The challenge becomes even more pronounced in multimodal GAI systems that integrate imaging, pathology, genomic information, and clinical records. Because the contribution of individual data modalities to the final prediction or recommendation remains difficult to determine, this limitation may reduce clinician trust and complicate accountability when AI-assisted decisions contribute to unexpected outcomes (115–117).

Emerging strategies aim to improve interpretability by incorporating external knowledge sources, structured reasoning frameworks, and evidence-grounded generation (64, 117, 118). For example, RAG approaches can constrain model outputs using curated clinical guidelines and literature (64), while knowledge-guided frameworks may enhance the alignment between AI predictions and clinically meaningful concepts (118). However, these approaches remain under active development and cannot fully resolve the inherent complexity of foundation models. Therefore, GAI should currently function as an assistive technology that provides transparent, evidence-supported outputs under appropriate clinical oversight rather than replacing clinician judgment.

### Data privacy and security

The application of GAI in lung cancer care raises important privacy concerns due to the use of highly sensitive and multidimensional clinical data. Lung cancer management involves the integration of longitudinal information, including radiological imaging, pathology findings, molecular profiles, treatment histories, and follow-up records. In particular, genomic alterations such as EGFR mutations and ALK rearrangements represent highly sensitive biological information. These data may introduce additional privacy risks even after conventional de-identification (119).

Unlike traditional clinical AI systems, many GAI rely on cloud-based infrastructures, raising concerns regarding data transmission, storage, secondary use, and potential exposure of patient information. These issues are particularly relevant when GAI is used to process clinical notes, imaging reports, pathology summaries, or molecular testing results in routine practice (120–122).

Ensuring secure and responsible deployment requires robust data governance, including privacy-preserving technologies, controlled access, transparent data usage policies, and appropriate regulatory oversight. Emerging approaches such as institution-specific deployment and federated learning may help reduce unnecessary data sharing while maintaining model utility (122–124). Establishing effective privacy protection frameworks will be essential for building trust and enabling the safe integration of GAI into lung cancer care.

### Evaluation and validation gaps

The safe and effective implementation of GAI in lung cancer care requires robust, clinically meaningful evaluation frameworks. However, current assessment approaches remain heterogeneous and insufficient for determining real-world clinical utility (10). Unlike conventional AI systems designed for specific prediction tasks, GAI applications in lung cancer encompass diverse functions, including clinical reasoning, guideline interpretation, report summarization, patient communication, and multimodal information integration. Therefore, evaluation strategies based solely on conventional performance metrics may not adequately capture their clinical value and potential risks.

A key challenge is the lack of standardized, task-specific evaluation frameworks. For diagnostic and prognostic applications, quantitative metrics such as accuracy, sensitivity, specificity, and AUROC remain important. Beyond these conventional metrics, GAI-assisted clinical reasoning requires additional assessment of factual correctness, guideline concordance, completeness, consistency, and safety (25). Recent studies evaluating LLMs in lung cancer screening and management have demonstrated that although models can generate clinically plausible responses, errors may occur in complex decision-making scenarios, including pulmonary nodule management and biopsy recommendations (49). These findings highlight that fluent language generation and medical knowledge alone do not ensure clinical reliability, emphasizing the need for rigorous and clinically relevant evaluation.

Beyond retrospective benchmarking, prospective clinical validation remains limited. Most current studies evaluating GAI in lung cancer involve simulated cases, retrospective datasets, or single-center analyses. Relatively few investigations have assessed its impact on clinical workflows, treatment decisions, or patient outcomes (49, 50, 62, 66, 75, 96). Future studies should incorporate external validation across diverse healthcare systems and patient populations, as well as clinically relevant endpoints to determine whether GAI improves diagnostic efficiency, decision quality, or patient care.

Furthermore, the continuously evolving nature of foundation models creates additional challenges for validation. Model updates, fine-tuning strategies, and changes in training data may alter performance over time, requiring ongoing monitoring rather than one-time validation. Establishing standardized reporting frameworks, longitudinal evaluation strategies, and multidisciplinary collaboration among clinicians, AI researchers, and regulators will be essential to ensure reliable, transparent, and clinically beneficial GAI systems in lung cancer care.

### Integration into clinical workflow

Despite the rapid advancement of GAI in lung cancer care, its integration into routine clinical workflows remains a substantial challenge. The multimodal and multidisciplinary nature of lung cancer management creates substantial challenges for data integration, workflow adaptation, and reliable implementation in real-world clinical environments.

A major barrier is the effective integration of GAI systems into existing healthcare infrastructures and clinical workflows. Unlike conventional AI systems developed for specific tasks, GAI applications in lung cancer often require the integration of heterogeneous and longitudinal information, including thoracic imaging, pathology, molecular profiling, treatment history, and clinical records. However, these data are often stored in heterogeneous formats across different clinical systems, limiting seamless information exchange and comprehensive AI-assisted analysis (125). Interoperability standards such as HL7 FHIR may facilitate structured data sharing and support the integration of GAI into clinical information systems (126, 127). In addition, deployment strategies such as institution-specific models or cloud–edge–terminal architectures may help balance computational requirements, data security, and regulatory considerations (128).

Beyond technical factors, successful adoption of GAI requires careful consideration of clinical workflow and human–AI interaction. In lung cancer care, GAI outputs must be delivered at appropriate clinical decision points and presented in a form that is interpretable and actionable for different healthcare professionals, including thoracic surgeons, oncologists, radiologists, and pathologists. Poorly integrated systems may increase cognitive burden rather than improve efficiency, while unclear accountability for AI-assisted decisions may limit clinician acceptance (129).

Therefore, GAI should be developed as an assistive technology that enhances rather than replaces clinical expertise. Future implementation will require multidisciplinary collaboration, clinician-centered system design, standardized deployment frameworks, and prospective evaluation of real-world clinical impact to ensure that GAI can be safely and effectively integrated into lung cancer care.

**Figure 3:**
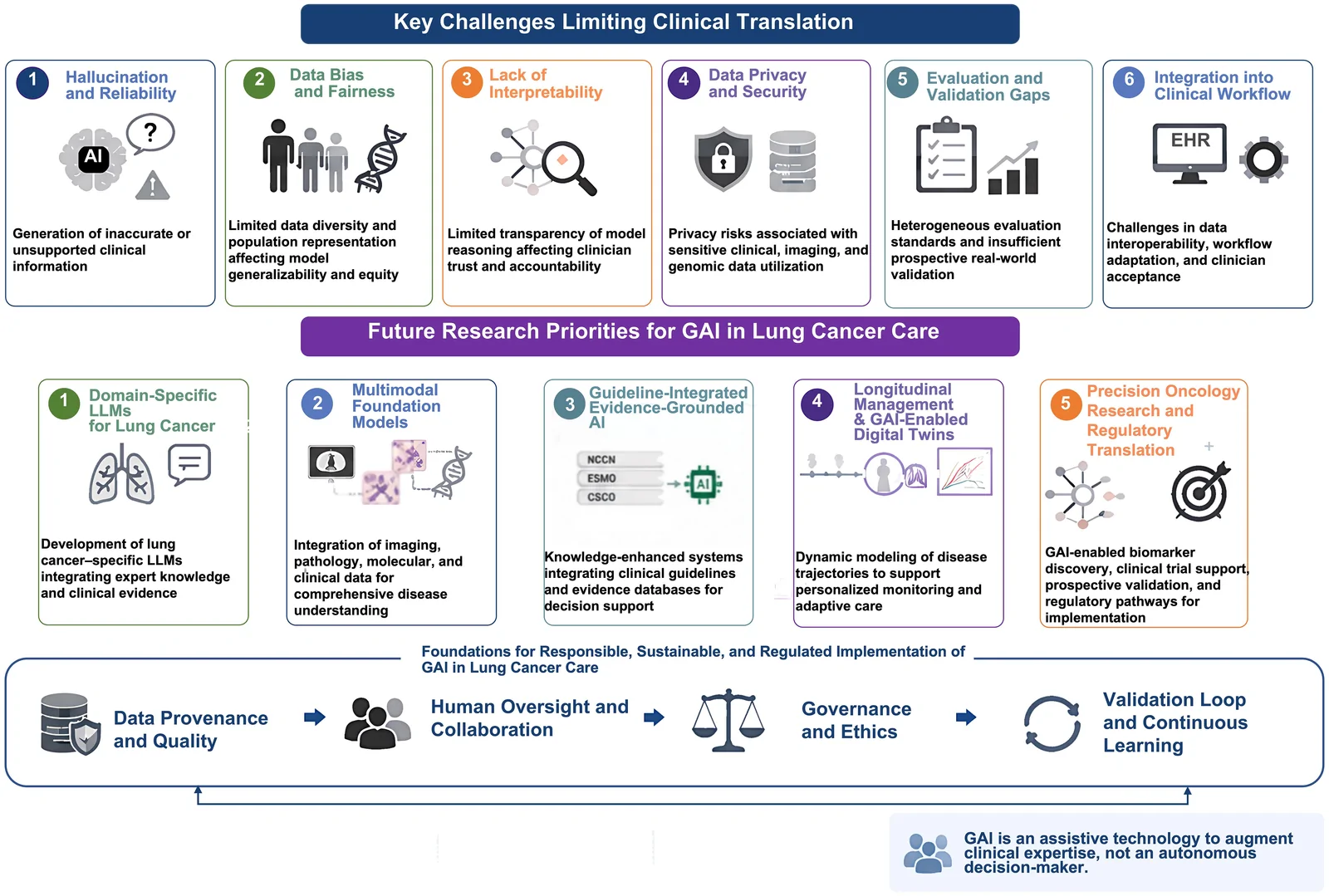
Challenges and future directions of GAI in lung cancer care. This figure summarizes the major barriers limiting the clinical translation of generative artificial intelligence (GAI) in lung cancer care and outlines future research priorities to advance its responsible implementation. Current challenges include unreliable outputs caused by hallucination, data bias and limited generalizability, insufficient interpretability, privacy and security concerns, heterogeneous evaluation standards, and difficulties integrating GAI into clinical workflows. Future research priorities focus on the development of lung cancer-specific LLMs, multimodal foundation models integrating imaging, pathology, molecular, and clinical data, guideline-integrated evidence-grounded AI systems, longitudinal management and GAI-enabled digital twins, and precision oncology applications supported by clinical validation and regulatory translation. The figure further highlights essential foundations for responsible GAI deployment, including high-quality and traceable data provenance, human oversight and multidisciplinary collaboration, governance and ethical frameworks, and continuous validation loops incorporating real-world monitoring and feedback-driven improvement. Together, these elements emphasize that GAI should function as an assistive technology to augment clinical expertise rather than replace clinician decision-making. GAI, generative artificial intelligence; LLM, large language model; EHR, electronic health record; RAG, retrieval-augmented generation; NCCN, National Comprehensive Cancer Network; ESMO, European Society for Medical Oncology; CSCO, Chinese Society of Clinical Oncology. Source: This figure was created by the authors and does not contain previously published material requiring permission.

Despite the significant potential of GAI in lung cancer care, several challenges remain that limit its safe and effective clinical implementation. These challenges include model reliability, data diversity and governance, interpretability, evaluation standards, privacy protection, and integration into clinical workflows. As illustrated in **Figure 3**, addressing these challenges requires more than improving model performance alone. It also depends on robust data foundations, human oversight, appropriate governance, and continuous validation throughout the clinical lifecycle. These considerations highlight several important directions for future research and clinical translation.

## Future directions

GAI is expected to reshape lung cancer care by progressing from isolated task-based applications toward integrated clinical support systems that can synthesize complex, longitudinal patient information (10, 25). Current studies have demonstrated promising capabilities of GAI in areas such as imaging interpretation, pathology information extraction, molecular profiling, treatment support, and patient communication. However, most investigations have evaluated general-purpose models within individual clinical scenarios and under controlled conditions. As illustrated in **Figure 3**, future development will focus on domain-specific, multimodal, and evidence-grounded GAI systems that integrate radiological, pathological, genomic, and clinical data to support more comprehensive lung cancer management. These systems may enable longitudinal disease management and precision oncology applications, while their clinical adoption will require robust validation, governance, and responsible implementation frameworks.

The development of domain-specific LLMs tailored to lung cancer care represents an important future direction. General-purpose LLMs have broad knowledge capabilities but may lack sufficient specialization for complex oncology scenarios. Early studies suggest that lung cancer–focused LLM approaches may improve performance in specialized tasks, including pathological information extraction, TNM staging, molecular interpretation, and clinical evidence synthesis. For example, lung cancer-oriented LLM systems have demonstrated improved accuracy and completeness in generating structured pathological databases compared with general-purpose models (73). Domain-adapted LLM approaches have also shown promise for automated TNM staging from radiology reports in lung cancer (56). Future domain-specific models may integrate expert-curated datasets, guideline-based knowledge, and continuously updated clinical evidence. Such models may provide more reliable and clinically relevant support for lung cancer practice.

Another major future direction is the development of lung cancer–specific multimodal foundation models. These models aim to integrate heterogeneous clinical information, including thoracic imaging, histopathology, genomic profiles, and longitudinal clinical records. Lung cancer diagnosis and treatment decisions inherently require the synthesis of multiple data sources rather than interpretation of isolated findings. Recent studies have provided early evidence supporting this transition. For example, multimodal large language models have demonstrated the ability to extract clinically relevant pathological features from lung adenocarcinoma specimens and use these features for downstream prognostic modeling (75). These findings suggest that GAI may function not only as a reporting tool but also as a bridge between pathological interpretation and clinical decision-making. Similarly, emerging PET/CT-based multimodal frameworks have shown potential for automated TNM-related information extraction and structured report generation in lung cancer cohorts, indicating the feasibility of integrating imaging analysis with language-based clinical reasoning (59). Such systems may therefore move beyond conventional single-modality AI systems by providing unified representations of radiological, pathological, molecular, and clinical information.

The integration of GAI with clinical guidelines and evidence-based frameworks represents another critical area for future development. Although current LLMs can summarize medical knowledge, their ability to independently apply complex guidelines remains limited, particularly for scenarios requiring nuanced clinical judgment (80). Future systems may overcome these limitations through RAG and integration with authoritative knowledge sources, including NCCN, ESMO, CSCO guidelines, clinical trial databases, and structured lung cancer management pathways. For example, a recent study showed that a RAG-equipped LLM achieved improved accuracy in lung cancer staging when provided with guideline-based external knowledge. These results suggest that knowledge-enhanced GAI systems may serve as reliable tools to support guideline-based clinical decision-making in lung cancer care (64). Rather than replacing clinicians, these systems may serve as evidence retrieval and synthesis tools that improve consistency and efficiency in guideline-based care. However, these systems will require continuous updating to incorporate rapidly evolving evidence and guideline changes in lung cancer, particularly given the expanding landscape of molecularly targeted therapies, immunotherapies, and antibody–drug conjugates.

Another important direction is the transition from isolated clinical applications toward longitudinal lung cancer management platforms. Current research on GAI in lung cancer has primarily focused on evaluating models within specific clinical tasks, such as report summarization, guideline interpretation, molecular information extraction, and treatment decision support. However, lung cancer care is a continuous process involving screening, diagnosis, staging, treatment selection, response evaluation, and long-term surveillance. Future systems may integrate information across the entire disease trajectory and provide dynamic clinical support throughout the patient journey. For example, studies evaluating LLM-based extraction from lung cancer CT reports have demonstrated the ability to identify tumor size changes, metastatic status, and disease progression from longitudinal imaging documentation (55, 61). In addition, recent investigations have shown that LLMs can interpret serial PET/CT reports and assess immunotherapy response according to standardized criteria, highlighting their potential role in treatment monitoring (86). Such longitudinal capabilities may enable GAI systems to continuously update patient profiles and support personalized surveillance strategies.

Beyond longitudinal monitoring, a more ambitious future direction is the development of GAI-enabled clinical digital twins for lung cancer management (130). Unlike conventional predictive models that estimate predefined outcomes such as survival or recurrence risk, digital twin approaches aim to continuously model individual patient trajectories by integrating longitudinal electronic health records, imaging findings, molecular profiles, and treatment histories. Recent work has demonstrated that large language models can forecast future patient health trajectories from sequential clinical records, highlighting their potential to simulate disease evolution and treatment responses. In lung cancer, such systems may eventually enable dynamic prediction of disease progression, therapy resistance, and potential clinical events, thereby supporting personalized surveillance and adaptive treatment strategies. However, substantial challenges remain, including longitudinal data integration, model validation, interpretability, and regulatory oversight before clinical implementation.

Beyond clinical decision support, GAI may also facilitate precision oncology research workflows in lung cancer. Emerging studies have explored LLM-based systems for clinical trial prescreening in NSCLC (131). These approaches may improve eligibility assessment and reduce the manual screening burden. They may also enhance trial recruitment efficiency and facilitate more precise matching between patients and investigational therapies.

In addition, LLMs may support therapeutic discovery by mining large-scale biomedical literature and clinical case reports for drug repurposing evidence (132). These applications may complement conventional drug discovery pipelines and help generate novel therapeutic hypotheses. However, current applications remain primarily research-oriented. Safety-aware frameworks and rigorous validation are still required before broader clinical implementation.

Future clinical translation of GAI in lung cancer will require not only robust validation frameworks but also the establishment of dedicated regulatory pathways for lung cancer–related GAI applications (133, 134). Unlike conventional AI systems developed for predefined tasks, GAI models are dynamic, adaptive, and capable of generating complex multimodal outputs. These characteristics create new challenges for evaluation and oversight (135). Dedicated regulatory pathways for lung cancer–related GAI applications will therefore be needed to establish standards for safety assessment, transparency, accountability, and continuous performance monitoring (136). These considerations are particularly important when AI-assisted outputs may influence critical clinical decisions, including pulmonary nodule management, biopsy selection, surgical planning, systemic therapy selection, and immunotherapy monitoring. Prospective multicenter validation studies with clinically meaningful endpoints will be essential to determine real-world effectiveness and support regulatory approval. Ultimately, GAI should be integrated into multidisciplinary lung cancer workflows as an intelligent assistant rather than an autonomous decision-maker, enhancing the capabilities of thoracic surgeons, oncologists, radiologists, pathologists, and other specialists through evidence synthesis, information integration, and decision support.

In summary, the future of GAI in lung cancer care will likely be characterized by the emergence of domain-specific LLMs, multimodal foundation models, guideline-integrated decision support tools, longitudinal disease management systems, and regulatory-ready precision oncology applications. Continued advances in model development, clinical evaluation, and regulatory governance will be essential to ensure that GAI can be safely translated into routine thoracic oncology practice and ultimately improve personalized lung cancer care.

## Conclusion

GAI represents a promising advancement in lung cancer care by enhancing clinicians’ ability to integrate complex clinical information, synthesize evidence, and support personalized decision-making. However, current evidence indicates that GAI remains an assistive technology rather than an autonomous clinical decision-maker, with limitations related to reliability, data bias, interpretability, and insufficient real-world validation. Future progress will depend on the development of domain-specific and multimodal systems, supported by rigorous clinical evaluation and appropriate regulatory frameworks. Ultimately, the value of GAI in lung cancer will lie not in replacing clinical expertise, but in augmenting multidisciplinary care and enabling more efficient, precise, and patient-centered management.
